# Supplementary material for: Behavioral Activation through Virtual Reality for Depression: A Single Case Experimental Design with Multiple Baselines
Source: J Clin Med. 2022 Feb 25;11(5):1262. doi: 10.3390/jcm11051262 (PMC8911126; doi:10.3390/jcm11051262)
Supplement: Supplementary file 1 [file jcm-11-01262-s001.zip › jcm-1577105-supplementary.pdf]

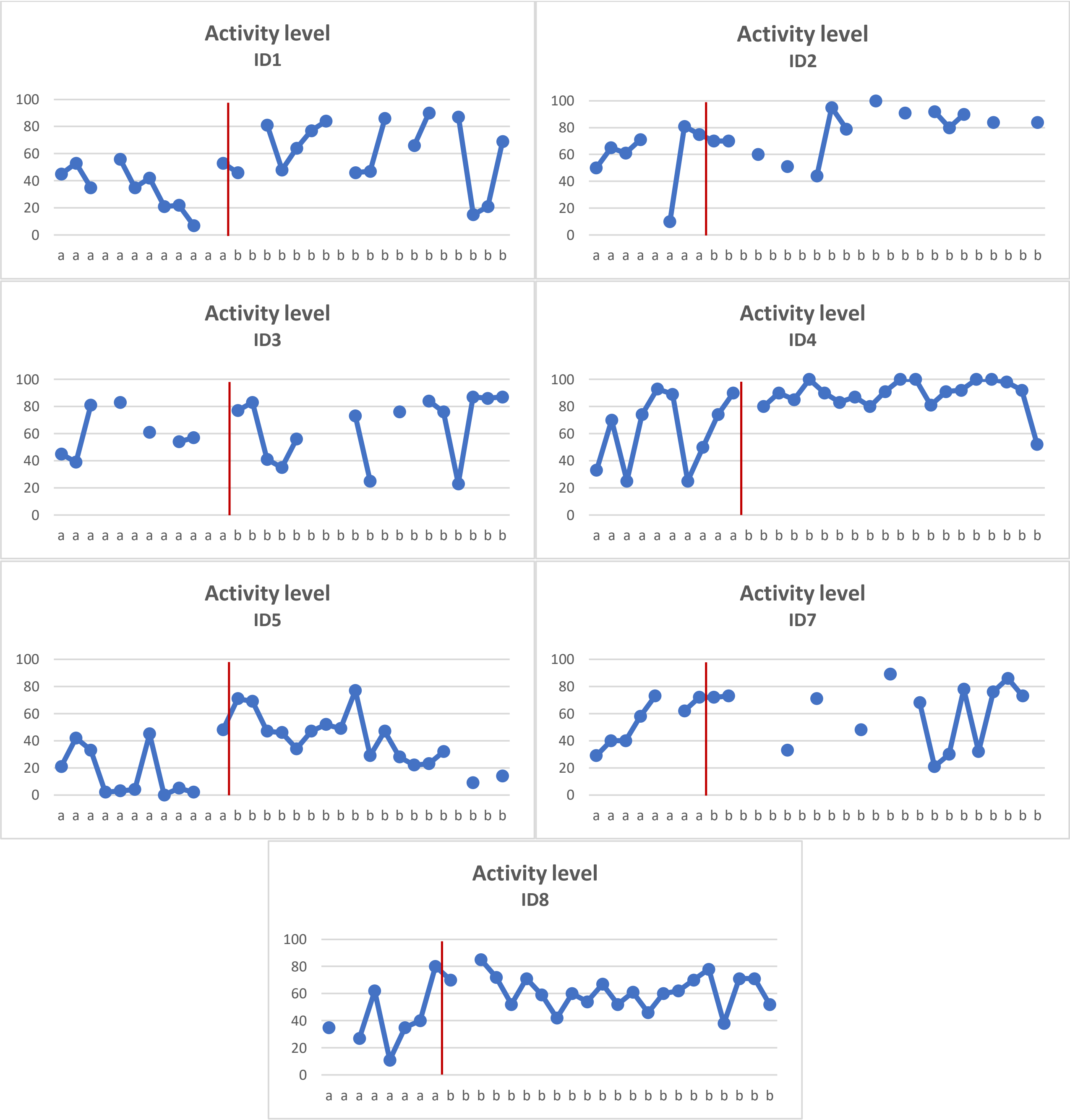

Figure S1. Activity level outcome graph: Visual inspection of patients' activity level measured through a 0-100 scale. a=baseline; b=intervention

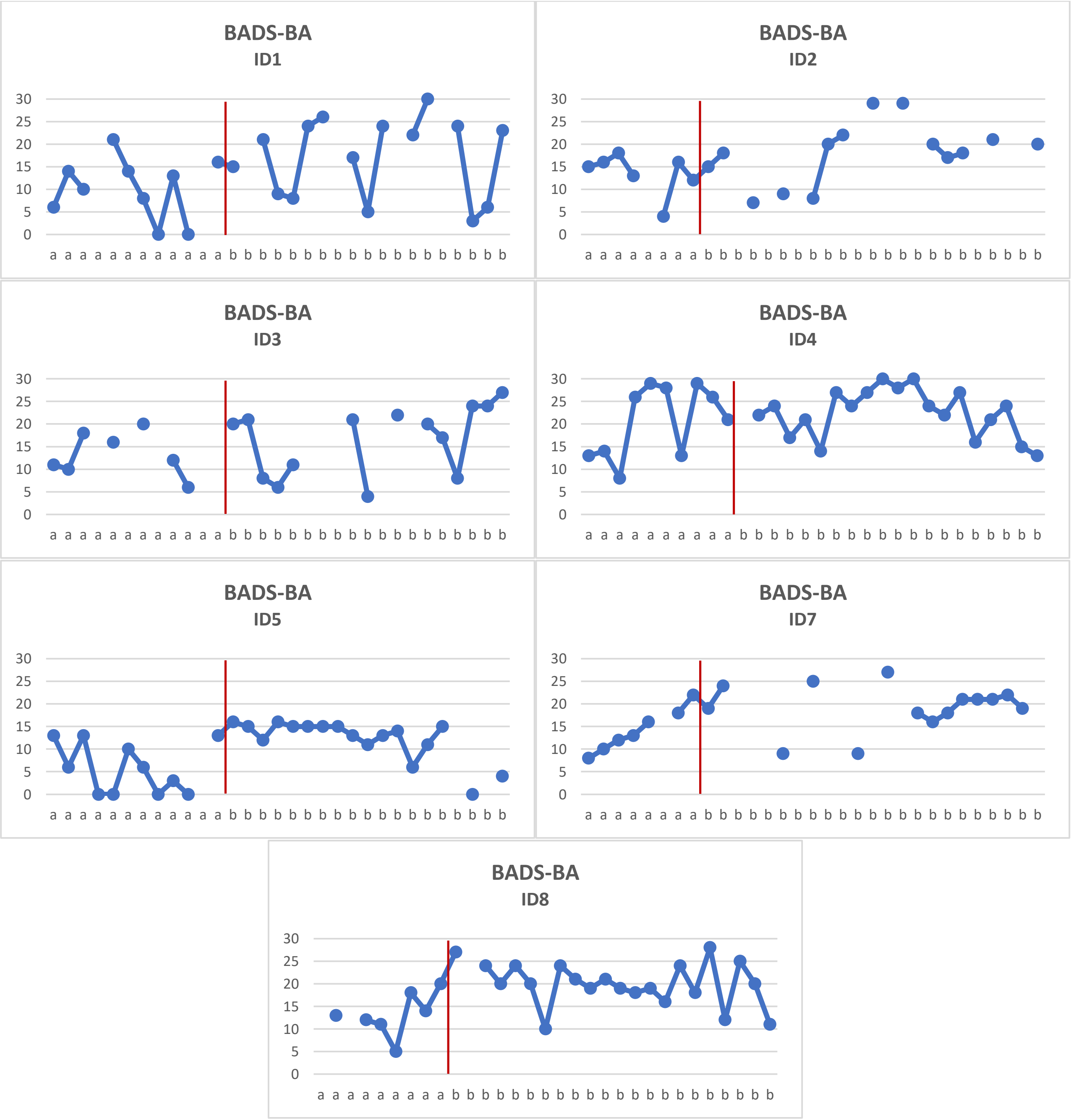

Figure S2. BADS-BA outcome graph: Visual inspection of patients' behavioral activation level measured through the Behavioral Activation for Depression Scale – Behavioral Activation subscale (BADS-BA). a=baseline; b=intervention

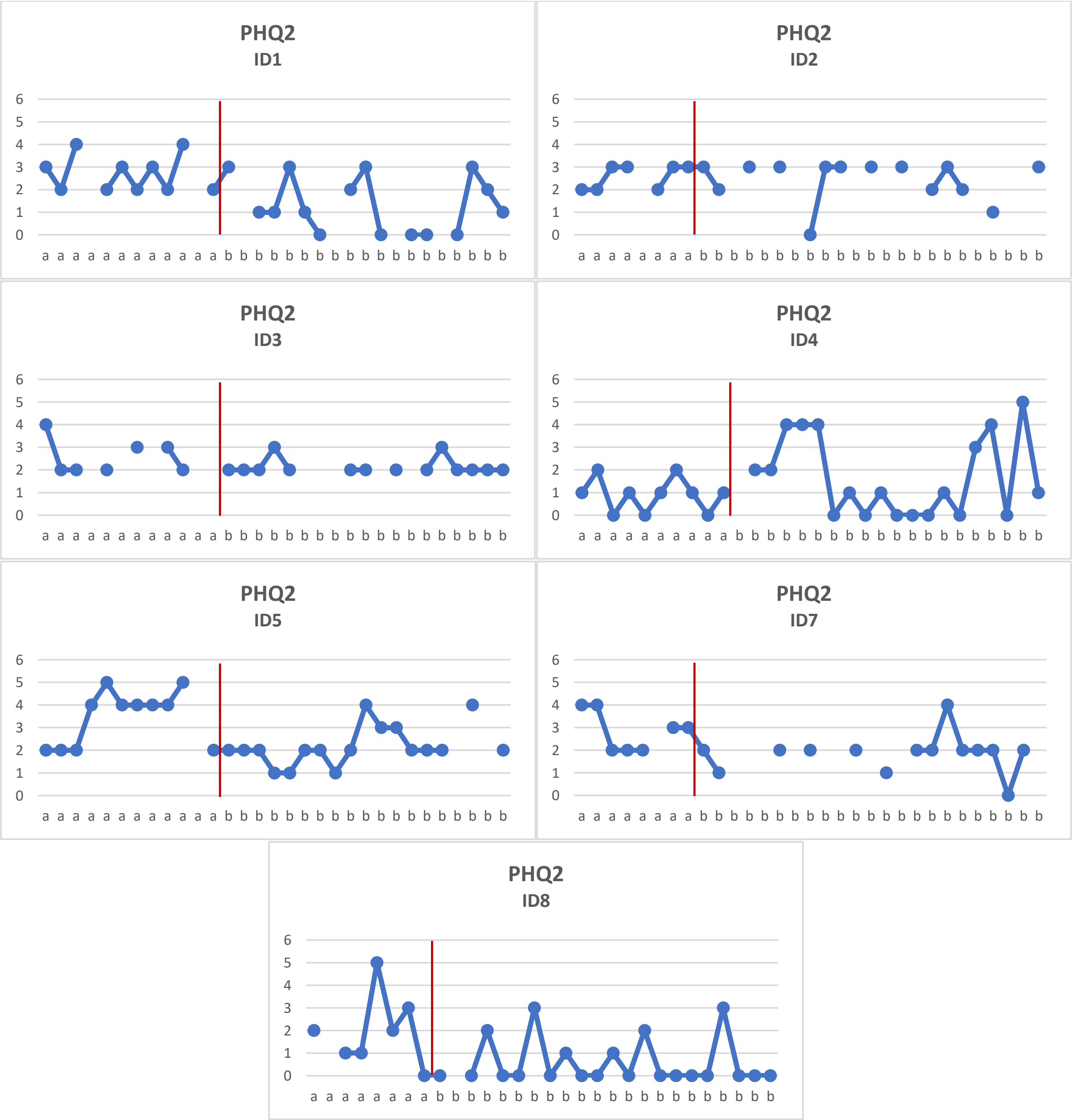

Figure S3. PHQ2 outcome graph: Visual inspection of patients' depressive symptoms measured through the Patient Health Questinnaire-2 (PHQ2).
a=baseline; b=intervention

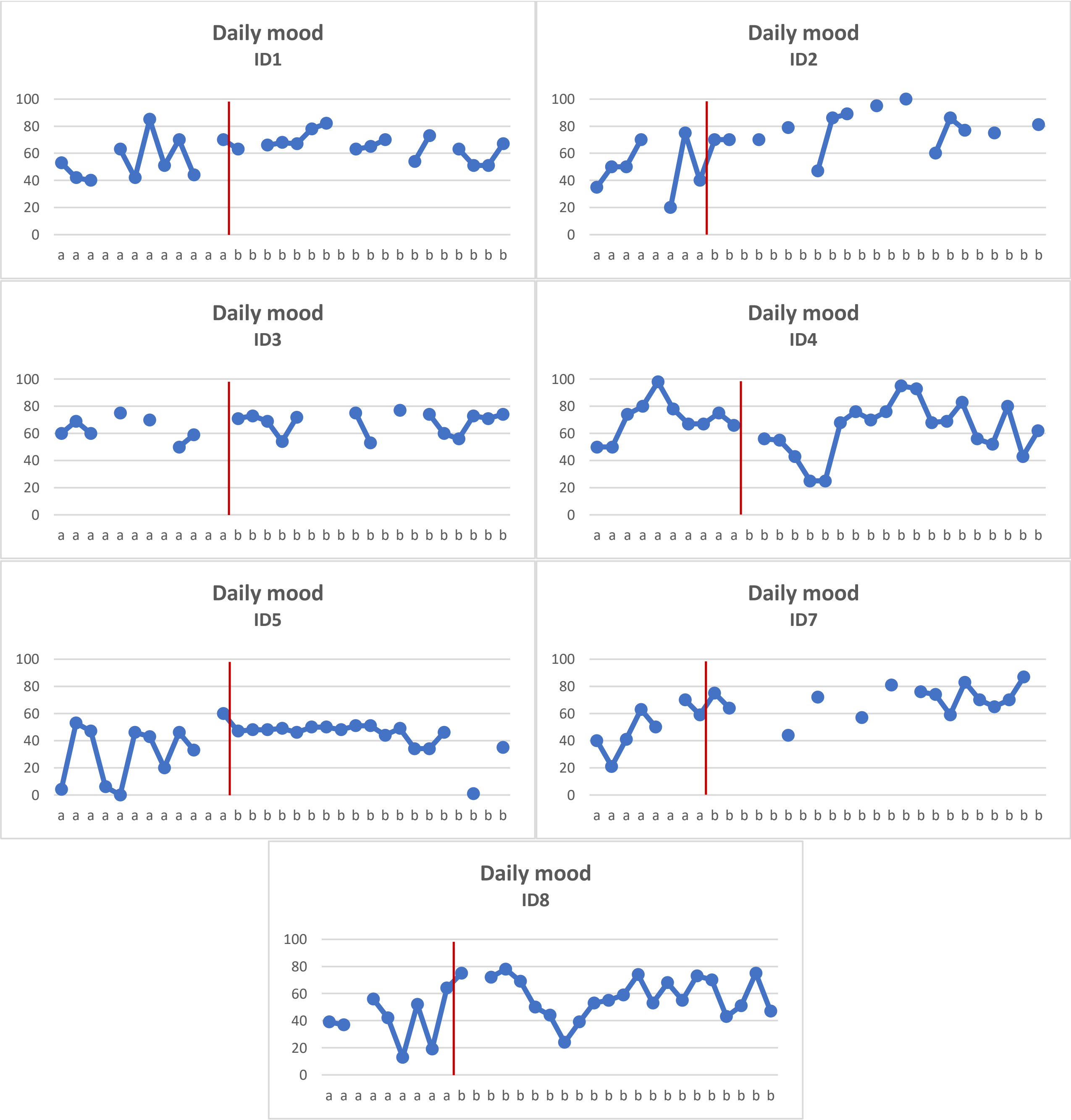

Figure S4. Daily mood outcome graph: Visual inspection of patients' daily mood measured thorugh a 0-100 scale. a=baseline; b=intervention

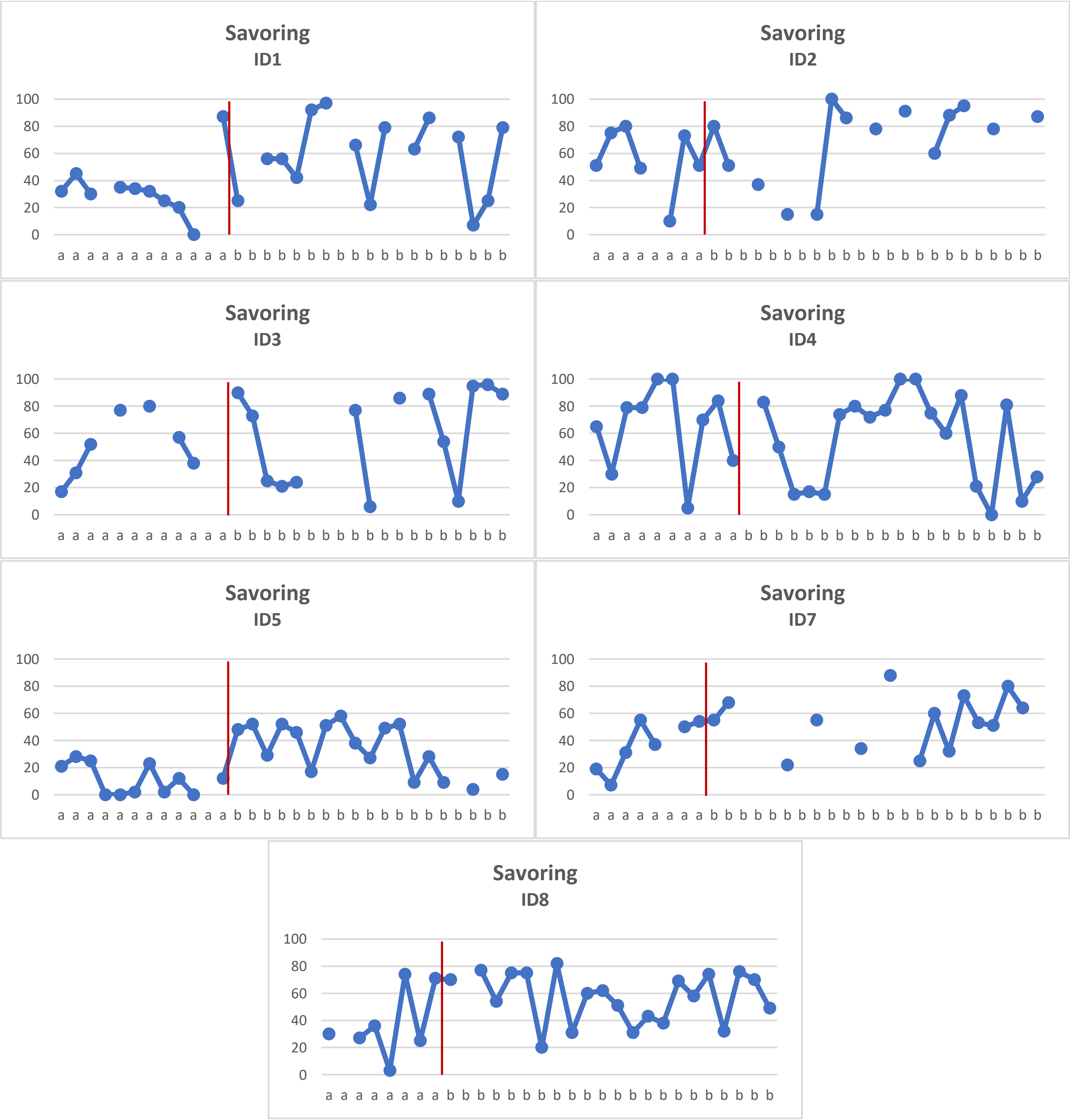

Figure S5. Savoring outcome graph: Visual inspection of patients' daily savoring measured thorugh a 0-100 scale. a=baseline; b=intervention
